# Airway Remodeling in Chronic Obstructive Pulmonary Disease and Asthma: the Role of Matrix Metalloproteinase-9

**DOI:** 10.1007/s00005-015-0345-y

**Published:** 2015-06-28

**Authors:** Katarzyna Grzela, Malgorzata Litwiniuk, Wioletta Zagorska, Tomasz Grzela

**Affiliations:** Department of Paediatrics, Pneumonology and Allergology, Medical University of Warsaw, Warsaw, Poland; Department of Histology and Embryology, Medical University of Warsaw, Chalubinskiego 5, 02-004 Warsaw, Poland; Potgraduate School of Molecular Medicine, Warsaw, Poland

**Keywords:** Asthma, Chronic obstructive pulmonary disease, COPD, MMP-9, Remodeling

## Abstract

Chronic obstructive pulmonary disease (COPD) and asthma are both associated with airflow restriction and progressive remodeling, which affect the respiratory tract. Among various biological factors involved in the pathomechanisms of both diseases, proteolytic enzymes—matrix metalloproteinases (MMPs)—play an important role, especially MMP-9. In this review, the authors discuss the current topics of research concerning the possible role of MMP-9 in both mentioned diseases. They include the analysis of protein levels, nucleotide polymorphisms of MMP-9 gene and their possible correlation with asthma and COPD. Finally, the authors refer to the studies on MMP-9 inhibition as a new perspective for increasing the effectiveness of treatment in asthma and COPD.

## Introduction

Chronic obstructive pulmonary disease (COPD) and asthma are two main diseases associated with airflow restriction, which affect the respiratory tract. Due to their high prevalence, they constitute a serious clinical and social problem. COPD is currently considered by the World Health Organization experts to be the fourth cause of death in the world and its frequency, especially its mild form, is assessed to be up to 25 % in adults above 40 years of age (Buist et al. 2007). According to epidemiological studies, the number of individuals affected by asthma is estimated at approximately 300 millions (Masoli et al. 2004; Sears 2014).

COPD is characterized by progressive, partially reversible, or irreversible, airway constriction. It is associated with chronic bronchitis, emphysema and pulmonary hypertension. The main etiopathogenic factor in COPD is chronic exposure to tobacco smoke; however, further deterioration may be associated with upregulation of chronic inflammatory response to various harmful constituents of air pollution (Global Initiative for Chronic Obstructive Lung Disease 2010).

Asthma is associated with bronchial constriction in response to intrinsic or environmental stimuli (Pascual and Peters 2005). Usually, the intensity and occurrence of clinical symptoms vary between individuals. The symptoms may regress during the course of treatment or spontaneously. Moreover, between episodes of aggravation and in remission, patients may not reveal any clinical signs of disease (Holgate 2007).

## Chronic Inflammation

There is no doubt that COPD and asthma are two distinct diseases with significantly different mechanisms of chronic inflammatory reaction. In COPD, the inflammation-associated changes are demonstrated predominantly in small airways and lung parenchyma, and result in tissue destruction with progressive, irreversible airflow restriction. The main changes in asthma are found in larger airways and may cause their intermittent and usually reversible obstruction (Broide 2008; Sethi et al. 2012). However, also in asthma, the airflow disturbances may be fixed, especially when involving the distal segments of the respiratory tract, in small airways, and when associated with significant thickening of the reticular basement membrane (RBM) and deposition of the extracellular matrix components (Broide 2008; Ward et al. 2002). It is noteworthy that the mentioned changes in RBM are considered as early features of airway pathology, which may be parallel or even primary to chronic inflammation (Ward and Walters 2005).

**Fig. 1:**
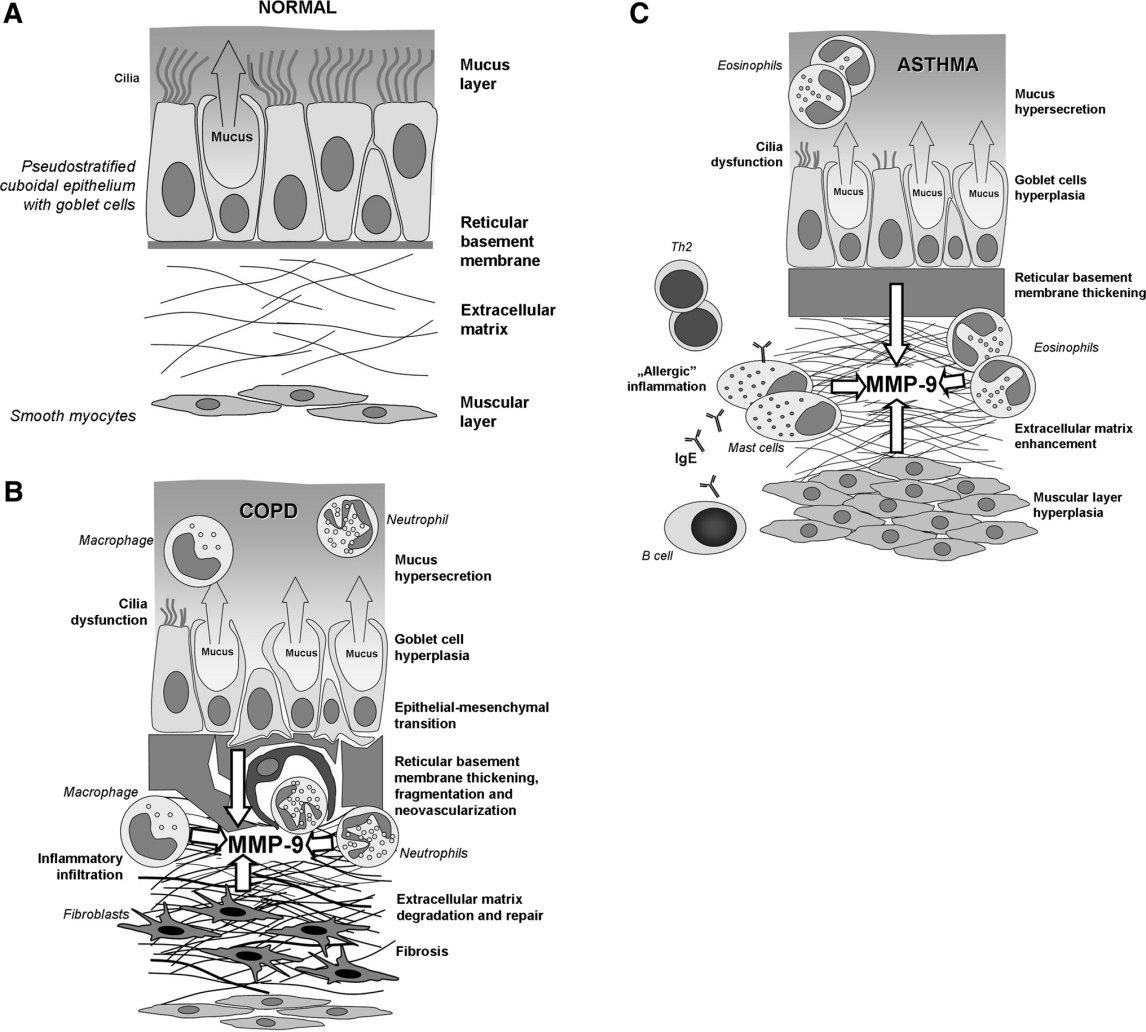
The schematic representation of airway mucosa: in a healthy individual (**a**), structural changes in COPD- (**b**), and asthma-associated remodeling (**c**). The detailed description is given in the text

The main cellular components of both COPD- and asthma-associated inflammatory reactions are also diverse. In COPD, in addition to epithelial cells, the main players are neutrophils and macrophages, whereas in asthma-associated inflammation eosinophils and mast cells predominate, with an increasing role of neutrophils during exacerbations (Fig. 1). On the other hand, some non-asthmatic patients may display eosinophilic bronchitis, resulting in fixed airflow obstruction, which is characteristic for COPD. Moreover, in some exacerbations, especially those associated with viral infections, increased neutrophils and eosinophils may be found in both COPD and asthma (Sethi et al. 2012).

It is an established dogma that each of the mentioned diseases is controlled by different immunoregulatory/immunocompetent cells. In COPD, mainly Th1 and CD8^+^ lymphocytes are engaged, whereas in asthma the key players are Th2- and IgE-producing B cells (Fig. 1) (Broide 2008; Sethi et al. 2012).

According to the diversity mentioned above, the main cytokines/chemokines also differ in both diseases. In COPD, the levels of “regular” pro-inflammatory markers—IL-8, tumor necrosis factor (TNF) and C-reactive protein—have been found to correlate with disease severity. At least in some cases, they may result from other co-morbidities, which are frequently seen in patients with COPD and are associated with systemic inflammation (e.g., diabetes, atherosclerosis, etc.) (Sethi et al. 2012). In contrast to COPD, in asthma, markers of “allergic” inflammation—interleukin (IL)-4 and IL-13—predominate; however, the role of IL-8 and TNF in the exacerbations has also been postulated (Broide 2008).

## Airway Remodeling

In addition to the above discussed inflammatory reactions, COPD and asthma are both characterized by progressive changes in respiratory tract, known as remodeling. In physiological conditions, this term concerns gradual structural changes of various tissues, which are necessary for proper formation and development during embryo- and organogenesis (Jeffery 2001b). However, in the pathophysiology of the respiratory tract, the remodeling concerns highly composed structural transformation, which affects the airways and leads to their significant functional impairment (James 2005).

There is no agreement on whether the airway remodeling is a consequence of the inflammation, or rather exists as a distinct phenomenon. As mentioned previously, the main trigger of inflammatory response in COPD is usually tobacco smoke or other inhaled constituents of air pollution (Jeffery 2001a). The prolonged or frequent exposure to pro-inflammatory agents may promote the development of chronic form of inflammation with damage and metaplasia of the respiratory epithelium (Grzela et al. 2013; Haswell et al. 2010; Lapperre et al. 2007).

It has been suggested that activated epithelial cells may change their phenotype to mesenchymal ones and this process is described as epithelial–mesenchymal transition (Kalluri and Neilson 2003). This observation is consistent with the concept of aberrant activity of “epithelial–mesenchymal trophic unit” (EMTU)—a structural and functional element in lung organogenesis (Holgate 2010; Ward and Walters 2005). Stimulated epithelial cells express large quantities of cytokines/chemokines and metalloproteinases. Thus, they possibly directly participate in thickening and also in fragmentation of RBM, characterized by formation of “clefts” (Sohal et al. 2014). The infiltration of small airways by neutrophils, macrophages and T lymphocytes is associated with further release of cytokines and proteolytic enzymes, resulting in the destruction of alveolar structure and increased mucus secretion (Sethi et al. 2012). The injured tissues tend to repair by a process similar to that of scar formation (Beckett and Howarth 2003; Pascual and Peters 2005; Ward et al. 2002). However, while exposure to noxious agents persists, actually this “repair” represents the outcome of two opposite processes, the enhanced proteolysis in stroma of pulmonary tissue and extensive fibrosis of the bronchial tree (Churg et al. 2009; Kranenburg et al. 2006; Salazar and Herrera 2011).

The airway remodeling occurs also in asthma, although localization and character of structural changes throughout the respiratory tract wall are different from those in COPD. The asthma-associated remodeling consists of RBM thickening, activation and proliferation of myofibroblasts and airway smooth muscle cells, and diffuse depositions of extracellular matrix components—collagen, fibronectin and proteoglycans (Mauad et al. 2007; Westergren-Thorsson et al. 2010). Moreover, important constituents of this remodeling include increased vasculature with angiogenic sprouting as well as the number and activity of epithelial goblet cells and mucous glands (Ward and Walters 2005). On the other hand, similarly to COPD, epithelial cells in asthmatics display increased expression of epidermal growth factor receptors, which may affect the proper control of epithelial cell cycles and their further behavior (Broide 2008; Hamilton et al. 2005). Interestingly, it has been reported that in some patients, RBM changes can occur even prior to clinical symptoms of asthma (Barbato et al. 2003). Therefore, the concept of epithelial–mesenchymal transition is exploited also in asthma (Holgate 2010).

As mentioned previously, the role of inflammation as a causative factor in asthma-associated remodeling is controversial. These controversies may be supported by observation that in some cases, chronic anti-inflammatory treatment with inhaled steroids does not completely prevent airway remodeling and has limited influence on the natural course of asthma in children (Bisgaard et al. 2006). Interestingly, it has been demonstrated that methacholine-induced bronchoconstriction may be sufficient to induce inflammation-independent airways remodeling in patients with asthma (Grainge et al. 2011). Although the detailed mechanism responsible for this phenomenon remains unclear, it may further support the role of EMTU-based concept in asthma.

COPD and asthma, although significantly different in their initial or stable stages, display more immunologic and molecular similarities in their severe forms and exacerbations. High local concentrations of pro-inflammatory cytokines/chemokines and fibrosis-promoting factors maintain the already initiated process (Decramer et al. 2012; Grzela et al. 2013; Rahman and Adcock 2006). A long list of key players involved in the process includes IL-8, TNF, fibroblast growth factor, insulin-like growth factor and, especially, transforming growth factor (TGF)-β (Holgate et al. 1999). TGF-β was found to stimulate collagen and fibronectin production by fibroblasts and, therefore, seems to play a crucial role in the induction of peribronchial fibrosis. Also, it seems to be involved in smooth muscle cells hyperplasia and mucus hypersecretion (McMillan et al. 2005; Panettieri et al. 2008; Xie et al. 2007). Simultaneously, it was demonstrated that TGF-β may inhibit the degradation of extracellular matrix components by the suppressive effect on collagenase expression and, on the other hand, the upregulation of natural tissue inhibitors of matrix metalloproteinases (TIMPs) (Wynn 2007). Interestingly, it has also been proven that TGF-β may reveal dual effect on MMP/TIMP balance, since in in vitro cultured human lung fibroblasts it stimulated the expression of both MMP-9 and TIMP-1 (Todorova et al. 2009). Therefore, it is plausible that airway remodeling followed by peribronchial fibrosis may result from a TGF-β-mediated mechanism similar to excessive repair.

## Matrix Metalloproteinases

Matrix metalloproteinases (MMPs) constitute a large family of Zn^2+^-dependent endoproteinases. So far, at least 25 distinct MMP family members have been identified and, based on their molecular structure, substrate specificity and mechanism of activation, classified into four groups—archetypal MMPs, matrilysins, gelatinases, and furin-activated MMPs (Fanjul-Fernández et al. 2010; Grzela et al. 2011; Hadler-Olsen et al. 2011).

MMPs are involved in extracellular matrix turnover and tissue repair (Crosby and Waters 2010). On the other hand, however, when out of control they may contribute to tissue destruction, as found in cancer metastasis, aortic aneurysm, delayed wound healing, etc. (Birkedal-Hansen et al. 1993; Klein and Bischoff 2011). Recently, it has been postulated that some MMPs, especially MMP-9, may also be involved in respiratory tract remodeling (Salib and Howarth 2003; Sampsonas et al. 2007).

MMP-9, or gelatinase B, is produced mainly by macrophages and neutrophils, but also by epithelial cells, mast cells, fibroblasts, and smooth myocytes (Abel and Vliagoftis 2008; Kimata et al. 2006; Liang et al. 2007). The overall scheme of the MMP-9 structure resembles those of other MMPs. It is composed of a propeptide sequence, the catalytic domain, fibronectin-like domain and hemopexin-like domain (Grzela et al. 2011). MMP-9 displays gelatinolytic, elastolytic and collagenolytic activity, thus playing a key role in extracellular matrix turnover. Due to its broad substrate specificity, in addition to cleavage of extracellular matrix components, MMP-9 may also modulate the activity of various biological factors, including other proteinases (e.g., MMP-13), their inhibitors (e.g., α1-antitrypsin) or cytokines (e.g., IL-1, VEGF) (Atkinson and Senior 2003; Engsig et al. 2000; Liu et al. 2000; Patterson and Sang 1997).

## MMP-9 in COPD and Asthma

Despite numerous studies conducted so far, current knowledge regarding the role of MMP-9 in COPD and asthma is still incomplete. It may, at least partially be due to methodological difficulties. To get the complete data, the measurement of the amount of protein, which can be easily done using simple ELISA, should be supplemented with some functional assay, reflecting biological activity of tested factor (Lowrey et al. 2008). In case of MMP-9, the method routinely used for its functional assessment is the standard zymography. This semiquantitative method is based on electrophoresis of tested samples in gelatin-enriched SDS/polyacrylamide gel, followed by gel incubation and visualization. Regrettably, the procedure does not allow the precise determination of active versus inactive MMP-9, since it is associated with uncontrolled activation of pro-MMP during electrophoresis (Grzela et al. 2011). Moreover, this SDS-mediated activation is difficult to predict and, therefore, may significantly impair the accuracy of assessment in general. Therefore, studies based on results from the standard zymography alone do not provide data sufficient for appropriate conclusions and should be accompanied by alternative in vitro assays, which allow the assessment of both, activity and amount of MMP-9 (Grzela et al. 2011). The principle of these assays is to detect the native enzyme in solution or after its immobilization by the specific antibody, followed by incubation with labeled substrate. Depending on the method used for substrate labeling, the detection system involves standard ELISA plate reader or more sophisticated equipment, e.g., real-time thermal cycler device (Grzela et al. 2014).

It is noteworthy that the sensitivity of these assays, which is similar to ultrasensitive ELISA, is even 20-fold higher, as compared to standard zymography. Therefore, they may be the best choice, especially in studies using material with the expected minute concentrations of MMP-9, e.g., in exhaled breath condensates (Grzela et al. 2015).

While reviewing studies conducted so far, elevated MMP-9 was found in blood, sputum and bronchoalveolar lavage from patients with asthma exacerbation (Cataldo et al. 2002; Gagliardo et al. 2009; Lee et al. 2001; Lemjabbar et al. 1999). In contrast, no such MMP-9 increase was found in patients with allergy, but without asthma (Belleguic et al. 2002). Another observation concerned the shifted ratio of MMP-9 to its natural inhibitor—TIMP-1—in bronchoalveolar lavage (BAL) fluid, which was higher in children with symptomatic asthma, as compared to that of healthy controls (Erlewyn-Lajeunesse et al. 2008). Murine models of asthma in MMP-9-deficient animals have confirmed the involvement of MMP-9 in peribronchial fibrosis. However, MMP-9-deficient animals still developed smooth muscle thickness, mucus hypersecretion and bronchial hyperresponsiveness (Lim et al. 2006).

Similarly to asthma-suffering individuals, also COPD patients displayed increased MMP-9 serum levels, which moreover correlated negatively with the Tiffeneau–Pinelli index (FEV1/FVC ratio) (Brajer et al. 2008; Erlewyn-Lajeunesse et al. 2008). The levels and activity of MMP-9 in sputum samples from COPD patients were found to be up to 12-fold higher, as compared to the control group. Both parameters correlated positively with neutrophil count and negatively with FEV1 % (of predicted value) (Culpitt et al. 2005). Surprisingly, some other group has shown that although sputum and BAL samples from COPD patients contained increased levels of MMP-9, nevertheless, their activity did not differ between groups (Lowrey et al. 2008). Furthermore, in the mentioned report, the authors have observed unexpected positive correlation between MMP-9 activity and FEV1/FVC ratio.

It is noteworthy that MMP-9 is thought to be one of the key executors in inflammatory reaction. In fact, increased levels of this protease were also found in acute respiratory tract diseases, including pneumonia of both bacterial and viral etiology (Kong et al. 2009; Brand et al. 2012). Therefore, MMP-9 level cannot be considered as a specific marker of COPD or asthma. However, the above studies provide some data, which may suggest its usefulness in monitoring airway remodeling in both diseases (Cataldo et al. 2002; Erlewyn-Lajeunesse et al. 2008; Gagliardo et al. 2009). Therefore, the diagnostic significance of this factor is still under clinical evaluation.

## MMP-9 Polymorphisms

An additional obstacle in MMP research may be the subtle variability in the molecular structure of the enzyme, which influences its specific activity. Such alteration may be determined by natural variation in the DNA sequence of the MMP gene. These changes, when occurring with frequency at higher than 1 % of the entire population, are identified as polymorphisms. Most commonly, the variability concerns single nucleotide in the gene sequence; therefore, such polymorphisms are known as single nucleotide polymorphisms (SNPs). The potential effect of individual SNP on protein structure and function depends on its character and position in the target gene structure. The majority of SNPs are functionally neutral; however, some of them may result in amino acid substitution, thus influencing the final protein structure, its biochemical properties, and, subsequently, physiological function. Therefore, numerous studies have been conducted so far to analyze the correlation between various pathologies and particular SNPs, including those in MMP-9 gene (Grzela and Bialoszewska 2010; Grzela et al. 2011; Ye 2000, 2006; Zhang et al. 1999). In fact, several SNPs identified in the MMP-9 gene were recognized as being associated with various diseases. Besides the reported correlation between some MMP-9 SNPs and cancer metastasis, aneurysm formation and atherosclerosis in coronary arteries and also their possible involvement in COPD and asthma have been postulated (Ganter et al. 2005; Holla et al. 2000; Pinto et al. 2010; Tesfaigzi et al. 2006).

The gene encoding for MMP-9 is located in chromosome 20 q13.12. It is composed of approximately 7.6 kilobase pairs, arranged in 13 exons (Collier et al. 1991). The first studies on the presumable role of MMP-9 polymorphisms were focused on SNPs located in the promoter region of this gene (Zhang et al. 1999). Subsequent research revealed at least four potential clinically relevant SNPs in the promoter, and at least another five, which have been found in the coding region (Grzela et al. 2011; Ye 2000, 2006). Nowadays, among over a dozen MMP-9 SNPs, the −1562 C/T SNP in the promoter sequence is the most frequently studied and best recognized polymorphism in the MMP-9 gene. Several reports suggested the importance of the common C/T substitution in the −1562 nucleotide of the MMP-9 promoter for gene transcription rate and protein level (Medley et al. 2004; Sampsonas et al. 2007). Interestingly, although recognized as risk factor in cancer metastasis, coronary artery disease, or aortic aneurysm formation, in COPD and asthma the mentioned SNP did not reveal any correlation with the prevalence and severity of diseases in Caucasian (Holla et al. 2000) or German populations (Ganter et al. 2005). However, two other reports suggested an association of MMP-9 −1562 T allele with higher susceptibility to COPD in non-Hispanic whites (Tesfaigzi et al. 2006) and Korean patients (Lee et al. 2010). None of the conducted studies have reported any correlation between other recognized SNPs in the promoter region, including −1831 T/A or −1702 T/A, and the susceptibility to asthma (Ganter et al. 2005).

While polymorphisms of the promoter region may influence the expression level of the target gene, the SNPs located within the coding sequence are potent modifiers of the biological properties of the final protein product. The *in silico* analysis of the putative impact of single amino acid replacement on the 3D structure and biological properties of the MMP-9 have suggested possible clinical relevance of several known polymorphisms of this enzyme. One of them concerns the 279th amino acid from the fibronectin-like domain of MMP-9 molecule, where the non-charged glutamine (Q) is replaced with a charged arginine (R). Thus, it is labeled as MMP-9 279 Q/R SNP. This particular SNP very likely affects the final structure of the enzyme and may result in its increased affinity to the substrate. The small study by Ganter et al. (2005) did not reveal any association between 279 Q/R SNP and asthma. However, subsequent studies in a large group of over 4000 children with asthma (Pinto et al. 2010) and COPD and suffering US veterans from New Mexico (Tesfaigzi et al. 2006) have shown a higher frequency of 279R allele in individuals affected by the mentioned diseases.

Another potentially functional MMP-9 574 P/R polymorphism is located in the hemopexin domain of the MMP-9 molecule. It was suggested that the substitution of proline (P) with arginine (R) in this SNP may attenuate the MMP-9 enzymatic activity. However, no relationship between the mentioned polymorphism and COPD or asthma and asthma-associated allergic rhinitis has been reported so far (Inoue et al. 2012).

## MMP-9 Modulators: the Future Perspectives?

The activity of MMPs is tightly controlled under physiological conditions by a number of natural factors. Apart from the specific TIMPs there are several other molecules, displaying MMP-attenuating properties. They include α2-macroglobulin, serpin E1/plasminogen-activator inhibitor-1, reversion-inducing cysteine-rich protein with Kazal motifs and tissue-factor-pathway-inhibitor 2 (Grzela et al. 2011; Litwiniuk et al. 2012). Moreover, several exogenous MMP modulators have also been developed. It is noteworthy that some of them are already used in clinical practice; however, their primary indication was different from the modulation of MMPs (Chakraborti et al. 2003; Fanjul-Fernández et al. 2010).

The first pharmacological interventions, directed against MMP-9 activity, have concerned synthetic proteinase inhibitors (e.g., batimastat, marimastat and ilomastat), originally aimed to prevent tumor metastases and tumor-related angiogenesis (Shono et al. 1998). However, due to numerous adverse events and relatively poor clinical effectiveness, they were not introduced to routine clinical use. Recently, natural MMP-9 antagonist, neovastat (AE-941), was found to reveal some beneficial properties in murine model of asthma (Lee et al. 2005).

Tetracyclines are natural antibiotics derived from *Streptomyces*. Besides their antimicrobial effects, tetracyclines are also able to inhibit MMPs activity by directly binding to their catalytic site. However, the studies on putative anti-MMP effects of tetracyclines are not consistent, and their inhibitory potential has not been fully confirmed (Curci et al. 1998; Ding et al. 2005).

The inhibitors of hydroxymethylglutaryl-coenzyme A reductase, widely known as statins, became recently a golden standard in the treatment of hypercholesterolemia and coronary artery disease. Besides their main hypolipemic action, statins, especially simvastatin and cerivastatin, reveal some poorly defined anti-inflammatory properties, including suppression of MMP-9 production in myocytes, neutrophils and macrophages (Nagashima et al. 2002).

The large group of potent MMP modulators was originally developed to regulate the function of the renin–angiotensin system in the management of arterial hypertension. However, the inhibitors of angiotensin-converting enzyme (ACE) are also well-known suppressors of MMPs activity, with a mechanism of action based on direct, dose-dependent blockage of the catalytic domain (Grzela et al. 2011). Another group of renin–angiotensin modulators comprises antagonists of angiotensin II receptor. They were shown to decrease the MMP expression, possibly due to suppression of the NF-κB pro-inflammatory pathways (Fujiwara et al. 2008).

Although the inhibition of the renin–angiotensin system may be considered as a novel therapeutic approach (Shrikrishna et al. 2012), nevertheless, determination of its clinical usefulness in COPD and asthma still requires further studies. This is especially important in the context of recent research concerning the insertion/deletion polymorphism of ACE (Ding et al. 2012), as well as the discovery of ACE2, the homolog of ACE (Kaparianos and Argyropoulou 2011). However, this issue is out of range of this review.

The airway remodeling in COPD and asthma is associated with the abnormal response of epithelium and other cellular components of the respiratory tract wall to noxious agents. There is still an open question, whether the remodeling results from inflammation or should rather be considered as parallel or even a primary process, which may precede clinical symptoms. Despite several differences, COPD and asthma reveal some resemblance, especially in their exacerbations and severe stages, where similar mechanisms and similar factors may be involved. Among various mediators involved in the progression of both the diseases are pro-inflammatory cytokines/chemokines, growth factors and proteases, including MMP-9. Moreover, there is growing evidence supporting the role of genetic predispositions in the development and progression of COPD and asthma. Therefore, better recognition of clinical relevance of the mentioned data may provide new opportunities for earlier diagnosis and more effective treatment.
